# Structural colour in *Chondrus crispus*

**DOI:** 10.1038/srep11645

**Published:** 2015-07-03

**Authors:** Chris J. Chandler, Bodo D. Wilts, Silvia Vignolini, Juliet Brodie, Ullrich Steiner, Paula J. Rudall, Beverley J. Glover, Thomas Gregory, Rachel H. Walker

**Affiliations:** 1 Division of Biosciences, University College London, Gower Street, London, WC1E 6BT, UK; 2 Adolphe Merkle Institute, University of Fribourg, Chemin des Verdiers 4, CH-1700 Fribourg, Switzerland; 3 Department of Chemistry, University of Cambridge, Lensfield Road, Cambridge, CB2 1EW, UK; 4 Natural History Museum, Department of Life Sciences, Genomics and Microbial Diversity Division, Cromwell Road, London, SW7 5BD, UK; 5 Royal Botanic Gardens, Kew, Richmond, Surrey, TW9 3AB, UK; 6 Department of Plant Sciences, University of Cambridge, Downing Street, Cambridge, CB2 3EA, UK

## Abstract

The marine world is incredibly rich in brilliant and intense colours. Photonic structures are found in many different species and provide extremely complex optical responses that cannot be achieved solely by pigments. In this study we examine the cuticular structure of the red alga *Chondrus crispus* (Irish Moss) using anatomical and optical approaches. We experimentally measure the optical response of the multilayer structure in the cuticle. Using finite-difference time-domain modelling, we demonstrate conclusively for the first time that the dimensions and organisation of lamellae are responsible for the blue structural colouration on the surface of the fronds. Comparison of material along the apical-basal axis of the frond demonstrates that structural colour is confined to the tips of the thalli and show definitively that a lack of structural colour elsewhere corresponds with a reduction in the number of lamellae and the regularity of their ordering. Moreover, by studying the optical response for different hydration conditions, we demonstrate that the cuticular structure is highly porous and that the presence of water plays a critical role in its ability to act as a structural light reflector.

By nano-structuring materials on the order of the light wavelength, it is possible to obtain colouration, termed structural colour, which, in contrast with pigmented colour, depends on structural characteristics rather than the chemical composition of materials^1^. Structural colour is widespread in nature^2^,^3^,^4^,^5^,^6^,^7^,^8^,^9^,^10^, and has been observed in a range of organisms, where it is employed for functions including visual communication, mate attraction and camouflage^11^,^12^,^13^. Photonic structures responsible for structural colouration have been well documented in underwater organisms^14^,^15^,^16^,^17^,^18^, land animals, including insects and birds^3^,^19^,^20^,^21^,^22^,^23^ and recently in plants^24^,^25^, within leaves^26^,^27^,^28^, flowers^29^,^30^,^31^ and fruits^32^,^33^.

The most common mechanism for structural colour production in nature is multilayer interference. Light propagating in such structures is reflected at interfaces between adjacent layers that differ in refractive index and can then constructively interfere^1^. Multilayer reflectors can be built from different materials including waxes and cellulose in plants^28^, chitin in insects^3^,^34^,^35^, keratin and melanin in bird feathers^36^,^37^,^38^ and guanine in fishes^39^. However, studies on taxa with structural colours that do not function in inter- or intraspecific visual communication significantly lag behind research on other structurally coloured taxa^11^. Few studies have researched structural colour in marine macroalgae and thus little is known about their structural mechanisms and functions.

Red algae (Rhodophyta) are one of the largest groups of algae with ~14,000 species estimated^40^. Cell structure and organisation differ between algal groups, species and even individuals. Two different mechanisms have been documented as the source of structural colouration: multilayer structures^41^ and iridescent bodies^42^,^43^,^44^,^45^,^46^,^47^,^48^,^49^,^50^,^51^. Iridescent bodies have been suggested to produce variation in the refractive index of light in comparison with the surrounding environment^52^,^53^. Since then, the majority of studies have focused on *Iridaea* spp. (Sethell & N.L.Gardner) P.C. Silva^54^ and *Chondrus crispus* (Stackhouse)^55^, showing that iridescent fronds contain a multilayered cuticle in contrast with their non-iridescent counterparts^41^,^56^. The *Chondrus crispus* life history contains two isomorphic phases, the gametophyte phase (when tissues have a haploid chromosome complement) and the tetrasporophyte phase (when tissues have a diploid chromosome complement)^57^. *Chondrus crispus* is present in intertidal and shallow subtidal zones, which in turn gives rise to the idea that structural colour may provide an adaptation to deal with environmental stresses such as UV or heat stress. Studies focusing on *Chondrus crispus* found that structural colour only occurred in the gametophyte stage of the lifecycle (Fig. 1a), and was never observed in tetrasporophytes. Craigie *et al.*^56^ observed relatively few cuticular layers (lamellae) in sporophytes compared with gametophytes, and showed that lamellae connect and fuse freely in sporophytes to form an irregular lamellae structure, potentially accounting for the lack of colouration in sporophytes. In all cases, colour was localised to 1.5 cm from the tip of the thalli^58^, although, the cause of this restricted distribution is not well understood. Gerwick and Lang^41^ were able to remove structural colouration by mechanically scraping off the cuticle layers, suggesting that the laminated cuticle could be involved in the production of colour. Taken together, these studies have identified structural features capable of producing colour, but lack both clear and quantitative elucidation of the mechanistic basis and an understanding of the biological purpose(s) of such colour.

Here, the experimentally measured optical response of *Chondrus crispus* fronds (Fig. 1) is correlated with their morphological structure by extensive finite-difference time-domain (FDTD) calculations using ultrastructural images from transmission electron microscopy. Sections from the tip, middle and base of fronds (Fig. 1b) are examined in order to compare differences in the morphological structure along the axis of the thallus. Lastly, the optical response of the frond is measured during dehydration to demonstrate the structural origin.

## Results

In all investigated fronds, structural colour was only observed in isolated patches at the tips of the thalli. The TEM images showed a significantly greater number of lamellae at the tip of the thalli (Fig. 2a) (M = 9.60, *SE* = 0.41, n = 30 (M, mean; SE, standard error; n, number of samples)) in comparison with the number of lamellae at the middle (Fig. 2b) (M = 2.6, *SE* = 0.26, n = 30; *t*(29) = 10.1, *p* < .0001), whereas at the base of the frond there was no evidence of a multi-layered structure (Fig. 2c). In addition, electron micrographs revealed that lamellae are more evenly deposited and consistently spaced at the tip of the thalli (Fig. 2a) in comparison with lamellae at sections from the middle of the thalli (Fig. 2b).

Optical measurements at the tip and base of the thalli support the role of the multi-layered structure (Fig. 3). With the optical microscope, a strong blue colour was only observed at the tip of the thalli (Fig. 3a), whereas the base of the thalli showed a dull brown colour (Fig. 3b). Furthermore, a peak in the reflectance spectrum at ~400 nm was seen at the tip of the thalli but no reflectance in the blue parts of the spectrum was observed at the base of the thalli (Fig. 3c).

We performed FDTD simulations based on the TEM images, including that of Fig. 2a, to gain optical insights. Results from these simulations showed that the reflectance spectra peak is in the blue, at ~460 nm, and are similar to the measured reflectance spectrum of the thallus tip shown in Fig. 3c, supporting the structural basis of the observed blue colour (Fig. 4b). The narrow spatial distribution of reflected light upon illumination (inset of Fig. 4b) further confirms the structural basis of the observed optical effect. Analytical modelling showed that an ideal multilayer with stepwise layer thicknesses, approximating the frond section, has the same reflectance (gray line in Fig. 4c). A model with the (hydrated) dimensions of the middle section of the frond (red line in Fig. 4c) showed no prominent peak due to less ordering and less layering of the cuticle.

Finally, in order to test the effect of hydration on the tissue we measured the reflectance spectra of *Chondrus crispus* thalli tips out of water, allowing the frond to dehydrate over time. As shown in Fig. 5, the presence of water in the system produced a peak in the reflectivity of UV – blue light (347–393 nm). However, as the tissue dried over time, a gradual loss in the intensity of the structural colouration was observed.

## Discussion

For the first time, our observations demonstrate that the structural colouration visible at the tips of *Chondrus crispus* thalli is due to constructive interference of light reflected by a multilayered cuticle structure. Furthermore, electron micrographs and spectroscopy data suggest that structural colour in *Chondrus crispus* is determined by the ability to achieve hydrated tissue and produce a high number of uniformly deposited cuticular lamellae.

A comparison of sections along the basal-apical axis of the frond reveals that the loss of structural colouration in older growth, at the base of a frond, results from a reduction in the number of lamellae as well as from degradation in the uniformity of layers. We suggest that the absence of cuticular layers in older growth is likely to be a result of a gradual degradation and shedding of layers without regeneration over time, rather than a lack of cuticular layer formation entirely in older growth. Our TEM images reveal a higher number of uniformly arranged lamellae at the tip of the thalli, in comparison with sections from the middle where only a double or triple layered structure is present and often not appropriately spaced for constructive interference to occur (Fig. 4c). Similarly, the optical response (Fig. 3) clearly demonstrates the intensity of reflected light confined to the tip of the frond. This structural colour, however, was not uniformly distributed, but varied locally between violet and green coloured patches (Figs 3a and 4b). This effect is most likely due to fine-scale heterogeneity of spacing in the multilayer structure (Figs 2a and 4b).

Within *Chondrus crispus*, the layered structure consists of electron-dense lamellae separated by less electron-dense interlamellar regions. Differences in the refractive index between lamellae and interlamellar regions are likely to be minimal and therefore require the absorption of water to increase the contrast in refractive index between the cuticular layers (Fig. S1). Previous studies have documented the role of water in the modification of structural colour. For example, iridescent feathers of mourning doves *Zenaida macroura* increased in overall reflectance by almost 50% following the addition of water^38^. The cuticle of *Chondrus crispus* is likely to be very similar to that of the related red alga *Iridaea* which is mostly composed of protein (50%) and carbohydrates (40%). Cuticular lamallae are associated with protein-rich regions and the interlamellar spaces with regions rich in carbohydrates^41^. Within *Chondus crispus*, these carbohydrates are rich in sulphated polysaccharides, which can contribute towards the retention of water and cause swelling, accounting for the large size increase in interlamellar regions following hydration. This is supported by the total loss of structural colour observed in *Chondrus crispus* as a result of dehydration, highlighting the dependence of this species on water to create the necessary contrast in refractive index between interfaces. Similarly, an examination of the layered structure responsible for structural colour in juvenile *Selaginella willdenowii* leaves showed that the required refractive index variation between interfaces stemmed from a difference in hydration^28^. This suggests that the role of water is a critical component for the production of structural colour in some species.

The reflectance of *Chondrus crispus* fronds is maximal in the ultraviolet to blue wavelength range, from ~320 to 430 nm (Fig. 5). Reflectance in these wavelengths may therefore function to protect against excessive levels of UV-blue light. Although individuals are likely to dehydrate and lose their structural colour within the intertidal zone, many individuals inhabit rock pools and thus remain hydrated with the potential to use structurally-based reflectance to reduce their overall levels of UV absorption. In contrast with phytoplankton that are capable of avoiding high exposure to light through vertical migration within the water column, marine macroalgae are mostly fixed and restricted to a single location. It is therefore likely that they have greater need for a mechanism capable of reducing excessive levels of UV radiation, especially in intertidal areas.

In some brown algae (Phaeophyceae) it has been shown that there is a direct relationship between the level of UV exposure and the number and viability of spores^59^. Thus hypothetically, structural colour in *Chondrus cripsus* may function specifically in gametophytes, rather than tetrasporophytes, to limit DNA mutations. Since the algal samples used for this study were collected from the intertidal zone of the shore, it seems plausible that structural colour may function as a protective measure for shallow growing gametophytes exposed to UV radiation for extended periods of time.

In conclusion, we have correlated the optical response of *Chondrus crispus* with a multilayered structure in the cuticle. By simulating the interaction of incident light with the multilayered structure, we demonstrate that cuticular lamellae are responsible for the structural colouration. Comparison of sections from the base, middle and tip of the thalli demonstrate that loss of the blue colour corresponds with reduction in the number of cuticle layers as well as degradation of their uniformity. In addition, we show that water is a critical component in the colour formation, providing the required contrast in refractive index between interfaces. Lastly, our results suggest an interesting hypothesis, that structural colour in *Chondrus crispus* functions to reduce the absorption of UV—blue radiation. This study furthers our understanding of structural colour in a poorly studied taxonomic group and paves the way for addressing questions of comparative evolution of iridescence in photosynthetic organisms, thus contributing towards an understanding of biological colouration as a whole.

## Methods

### Material Collected

**Figure 1:**
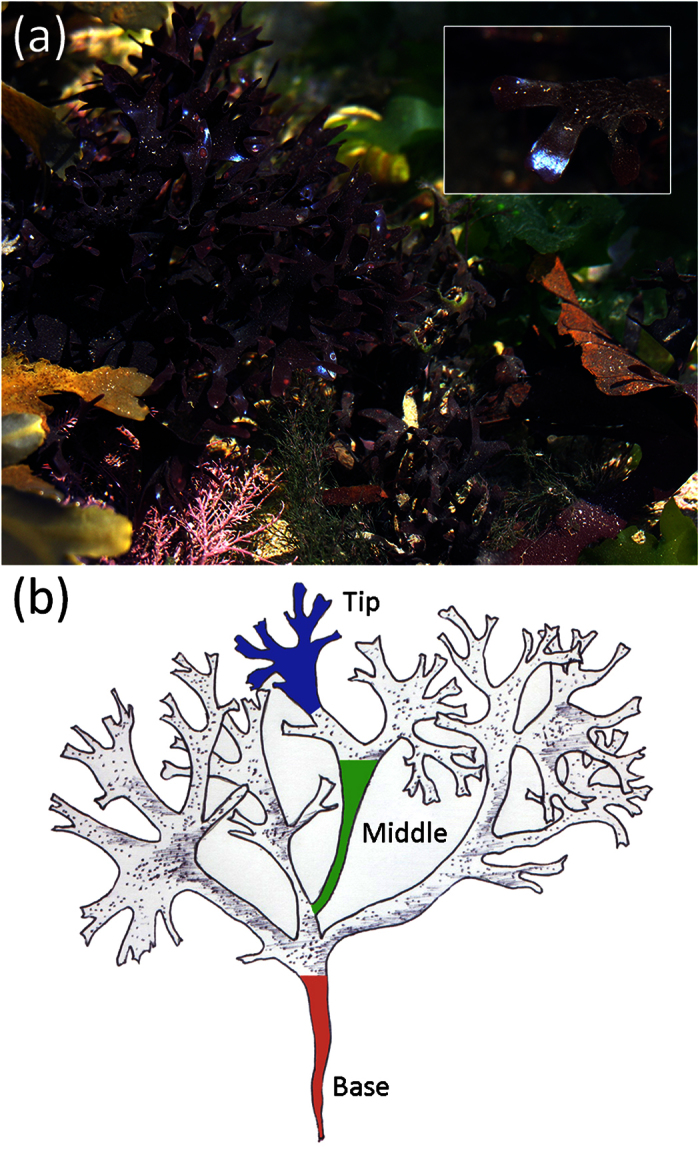
(**a**) Habitat view of Irish moss, *Chondrus crispus*. The intense blue colour at the tip of the thalli is clearly visible and strongly contrasts with the purple-red colour of the frond. (**b**) Illustration of a typical *Chondrus crispus* sample highlighting the position of the different sections of the thalli investigated in this study; base (red), middle (green) and tip (blue). All photographs in this figure were taken by Chris J. Chandler.

Samples of *Chondrus crispus* were collected from Peveril Point, Swanage, UK (50° 36’ 27.140” N, 1° 56’ 39.098” W) for Transmission Electron Microscopy (TEM) analysis. Structurally coloured gametophytic samples were randomly collected within the intertidal zone, ensuring that a representation of sections from the tip, middle and base of the thalli were obtained. Growth within the frond is apical, therefore the oldest tissue lies at the base and the youngest at the tip (Fig. 1b). Larger frond samples were also collected for spectroscopy analysis. The collected fronds were kept in a cooled environment (~12 °C) submerged in a distilled marine solution during transportation.

### TEM (Transmission Electron Microscopy)

Collected frond pieces were fixed in a 2.5% glutaraldehyde and filtered seawater mix followed by post fixation in a 1% Osmium tetroxide solution. Dehydrated samples were then washed in a 0.1 M Sørensen’s phosphate buffer (pH 7.2^60^) followed by dehydration in a graded ethanol:phosphate buffer series. Samples were taken through a graded ethanol:LR White Resin series before embedding and sectioning. Sections were cut from polymerised blocks using a Reichert-Jung Ultracut E ultramictrotome. Ultrathin sections (50–100 nm thick) were cut with a Diatome diamond knife. Sections were collected using Formvar coated copper mesh grids (Agar Scientific, UK). Sections were imaged on a Hitachi H-7650 TEM with integral AMT XR41 digital camera. A total of 258 images of cuticular lamellae were collected. Measurements of lamellae and cuticle thickness were obtained from 30 samples within each section of the thalli using the point to point measurement tool within the AMT Image Capture Engine v.600.355 software or ImageJ v.1.47^61^.

### Optical Imaging and Spectroscopy

Optical imaging was performed with an Olympus BX-51 optical microscope equipped with a 20X objective (Olympus, MPLFLN-BD 20x, NA = 0.5). The *Chondrus crispus* frond specimen was submerged in water and spectra were obtained at normal incidence in the range of 250–850 nm from the surface of the frond. These collected spectra allowed full observation from within the ultraviolet range, to demonstrate any potential photoprotective function, to within the near-IR range, highlighting any potential photosynthetically-enhancing absorption. A total of 150 spectra were recorded every five seconds for a total of 750 seconds under normal ambient conditions. Spectra were collected with a bifurcated reflection probe (Ocean Optics, BIF200-UV-VIS), with the imaging fibre connected to a deuterium-halogen light source (Ocean Optics DH-2000, 215–2000 nm) and the collection fibre connected to a spectrometer (Ocean Optics QE65000, 200–880 nm). The collected reflectance spectra were standardized with respect to a white Lambertian reflectance standard (Lab-sphere Spectralon SRM-99) in the case of the double-ended probe measurements. For the spectra collected using the microscope (as explained in^23^), we normalised the signal with respect to a protected silver mirror (Thorlabs, reflectivity up to 99% in the region 400–700 nm) in the optical microscopy measurements.

### Finite-difference time-domain (FDTD) modelling

Light scattering by the internal structure of the fronds was simulated using a three-dimensional finite-difference time-domain (FDTD) method for different ultrastructures obtained from TEM images. FDTD simulations can generally be used to calculate the light-matter interactions of arbitrary geometries. The use of FDTD modelling has a number of advantages over classic modelling methods, including allowing the input of measured TEM images with an assignment of a refractive index to each greyscale and calculating a spatial light scattering pattern reflected from that structure (see ref. 37). For the simulations we used Lumerical 8.7 (Lumerical Solutions Inc., Vancouver, Canada, http://www.lumerical.com/tcad-products/fdtd/), a commercial-grade simulator based on the FDTD method^62^.

The structural chemistry of the cuticle is not well characterised, thus the refractive index values of the material components are unknown. We therefore assessed the average refractive index of the algal frond from dried material by measuring the ratio of reflected light, 4%, and applying Fresnel’s formula, 
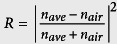
. The average refractive index thus derived is *n*_ave_ = 1.5. Assuming the non-stained material in TEM images to be composed of hydrated cellulose with a refractive index of *n*_Cellulose_ = 1.46^63^, the dark-staining material has an approximate refractive index value of ~1.55. In addition, cuticular lamellae in the algae frond change from the natural hydrated state when dehydrated for the TEM preparation. To model the optical response from the hydrated frond, we therefore have to rescale the dimensions of the TEM image. Gerwick and Lang^41^ suggest that the entire cuticle increases by 1.8 times its thickness when hydrated in water, specifically individual layers increase by 1.3 times in thickness and interlamellar regions increase by twice their thickness.

**Figure 2:**
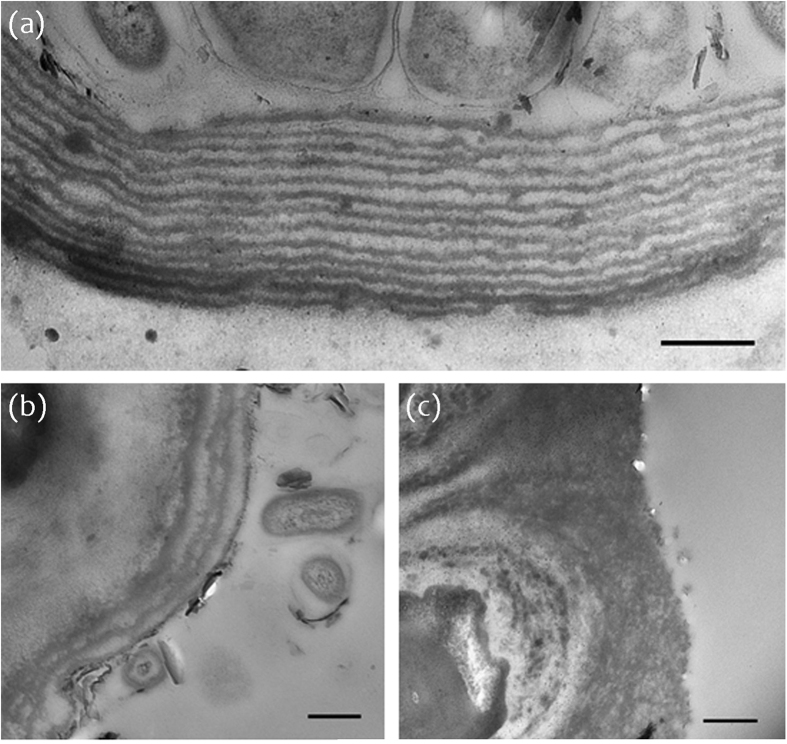
TEM micrographs showing the cuticle of a *Chondrus crispus* frond at sections from (**a**) tip, (**b**) middle and (**c**) base. Scale bars represent 500 nm (**a**,**b**), 2 μm (**c**); optical zoom, (**a**) 7000x, (**b**) 4000x, (**c**) 6000x.

**Figure 3:**
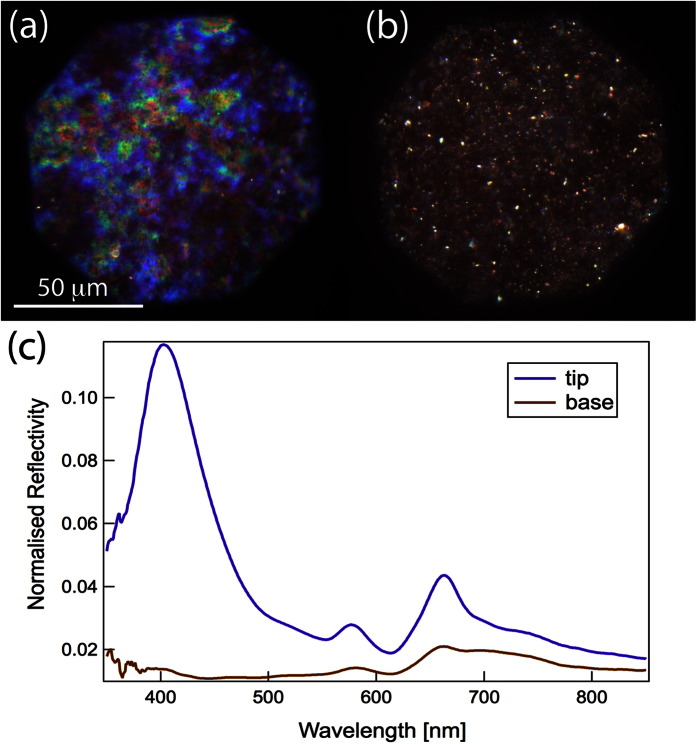
(**a**,**b**) Micrographs of *Chondrus crispus* at the (**a**) tip and (**b**) base of the thalli. (***c***) Reflectance spectra collected in confocal configuration under the microscope in the same area as images (**a**) and (**b**).

**Figure 4:**
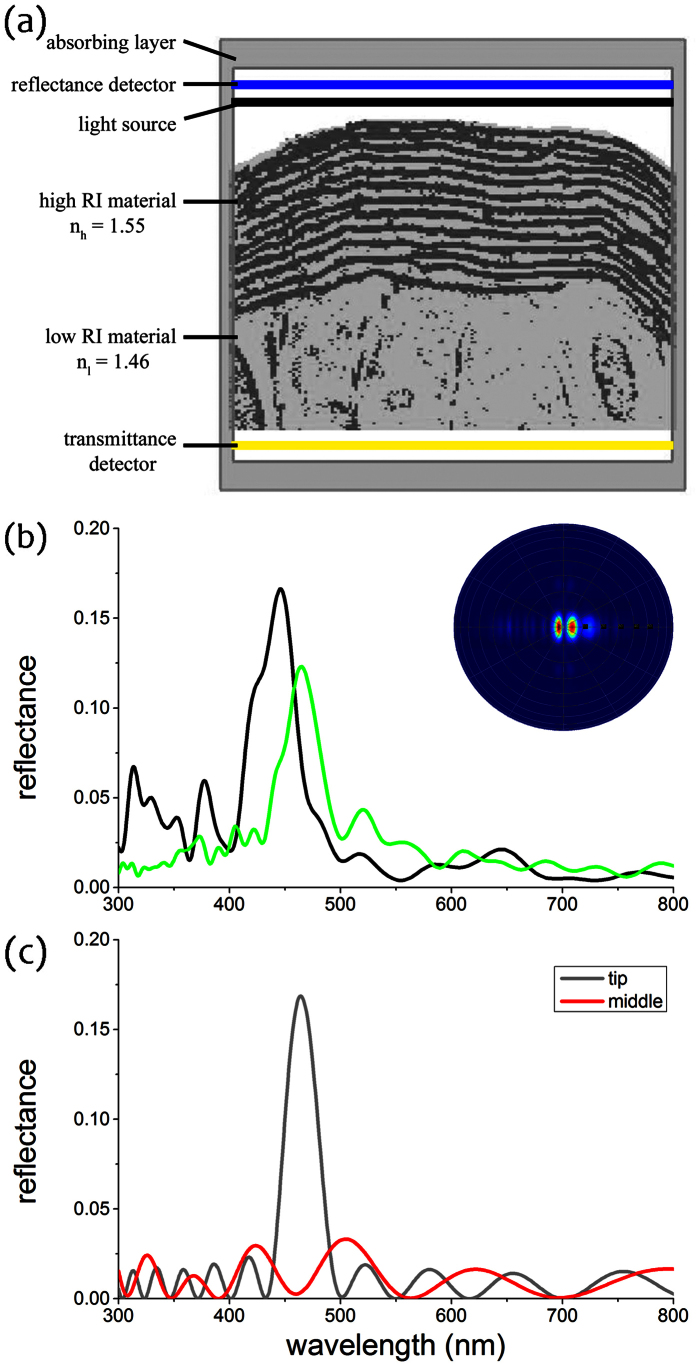
FDTD simulations. (**a**) Binarized version of the TEM image in Fig. 2a in the modelling volume assigned in the FDTD simulations. The light cellulosic material was assigned a refractive index of 1.46, whereas the dark-stained material was assigned a refractive index of 1.55. The boundary box was filled with water, RI = 1.33 (see also Materials & Methods). The outlined area indicates the computational domain with absorbing boundaries, the black bar the position of the light source and the blue and yellow bars the reflectance and transmittance detector, respectively. (**b**) Simulated reflectance spectra for non-polarized light at normal incidence from FDTD simulations of two different TEM images (green line for structure of Figs 2a and 4a; black line for structure of another TEM image). *Inset*: Simulated light scattering pattern shows a strong directionality of reflected light. (**c**) Reflectance spectra of an idealised classical multilayer model for the structure in the tip (gray line) and the middle of the frond (red line).

**Figure 5:**
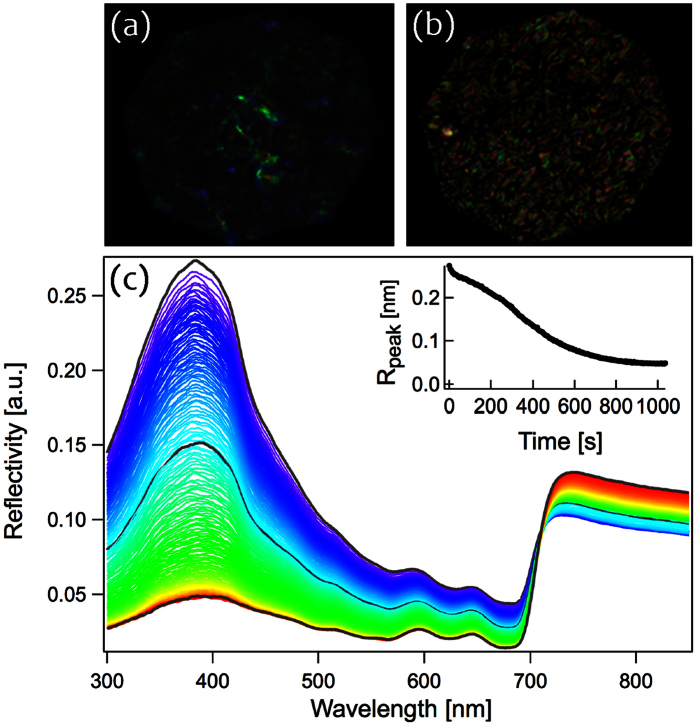
Loss of structural colour during the evaporation of water. (**a**) Wet tissue producing intense structural colouration, (**b**) dry tissue and loss of colour intensity. (***c***) Reflectance spectra of *Chondrus crispus* showing a reduction in peak reflectivity over time. Spectra were collected over 750 seconds at regular intervals of five seconds during the evaporation of the water. Different colour spectra represent different time intervals; wet tissue corresponds with blue spectra, dry corresponds with red spectra. Peak reflectance, 385 nm; measurements were performed under 0° incidence. *Inset:* the loss of colour intensity at the peak reflectance over time.

We performed FDTD simulations based on the TEM image in Fig. 2a, which was rescaled (as above) due to expected dehydration effects that occur in the TEM preparation. The modelling volume surrounding the algae frond was filled with water (RI = 1.33) to simulate natural aqueous conditions. The incident light beam was assumed to be a plane-wave with a broad-band spectrum covering 300–800 nm. The reflectance is subsequently calculated by the ratio of the light flux incident onto the structure and the light flux reflected through the detector plane above the frond (blue line in Fig. 4a). The spatial far-field light scattering profile is calculated by performing a far-field projection of the calculated field intensity in the same plane. We then simulated the reflectance spectrum for non-polarized incident light from a binarized model of the lamellar structure at the thalli tip (Fig. 4).

### Multilayer modelling

The reflectance of the multilayered structure in the algae frond was calculated by applying classical multilayer theory for dielectric media based on the transfer matrix approach using MatLAB^64^. For this, we simplified the frond ultrastructure of Fig. 2a into 16 equally spaced layers of 85 nm and 70 nm, with varying refractive index of 1.46 and 1.55 for the two repeating layers, respectively (see above). Water with a refractive index *n* = 1.33 surrounded the multilayer on both sides. To model the optical effect from the middle section of the frond, we calculated a layer profile with thickness 50 nm/140 nm/30 nm/90 nm/60 nm/600 nm with refractive indices 1.55/1.47/1.55/1.47/1.55/1.47, respectively.

### Statistical analyses

Student’s t-tests were performed to determine whether the variation between the mean number of cuticular layers was statistically significant between sections from the tip and middle of the thalli. Tests were conducted using the statistical software MINITAB, release 16 (Minitab Inc., State College, PA, USA). The *p*-value for statistical significance was set at 0.05.

## Additional Information

**How to cite this article**: Chandler, C. J. *et al.* Structural colour in *Chondrus crispus*. *Sci. Rep.* **5**, 11645; doi: 10.1038/srep11645 (2015).

## Supplementary Material

Supplementary Information
