# Supplementary material for: Structural colour in Chondrus crispus
Source: Sci Rep. 2015 Jul 3;5:11645. doi: 10.1038/srep11645 (PMC5155586; doi:10.1038/srep11645)
Supplement: Supplementary Information [file srep11645-s1.docx]

**Supplementary Material**


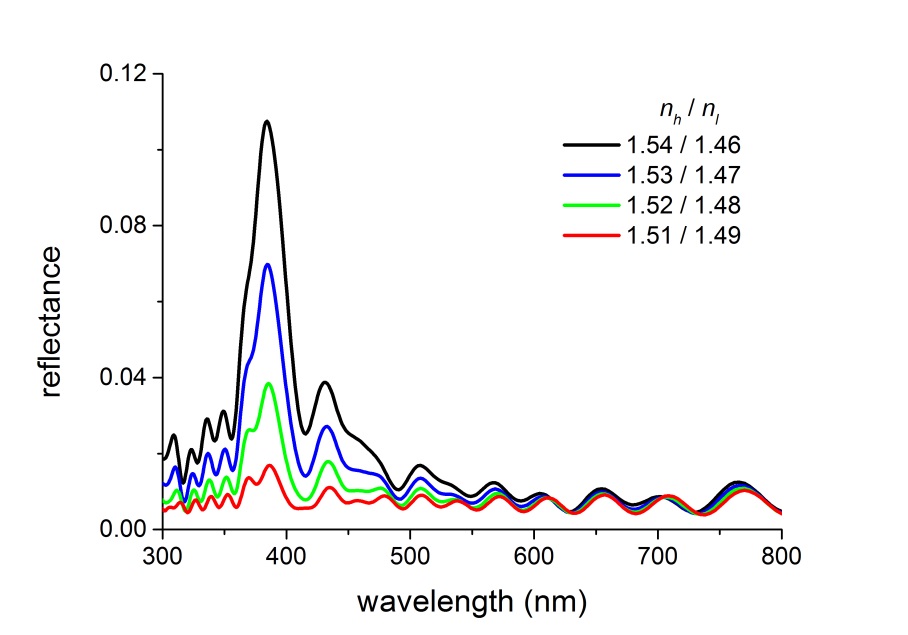


**Supplementary Figure 1.** Spectral patterns of the multi-layered structure with a lowering RI contrast between the layers using FDTD modelling.
